# Early detection and quantification of cerebral venous thrombosis by Magnetic Resonance Black Blood Thrombus Imaging (MRBTI)

**DOI:** 10.1186/1532-429X-18-S1-P16

**Published:** 2016-01-27

**Authors:** Qi Yang, Zhaoyang Fan, Xiaoming Bi, Debiao Li

**Affiliations:** 1 grid.50956.3f0000000121529905Biomedical Imaging Research Institute, Cedars Sinai Medical Center, Los Angeles, CA USA; 2 MR R&D, Siemens Healthcare, Los Angeles, CA USA

## Background

Early diagnosis of cerebral venous and sinus thrombosis (CVT) is currently a major clinical challenge. We proposed a selective MR black-blood thrombus imaging technique(MRBTI).

## Methods

MRBTI was performed on 23 patients with proven CVT and 24 patients with negative CVT by conventional imaging techniques. Signal-to-noise ratio (SNR) was calculated for the detected thrombus and contrast-to-noise ratio (CNR) was measured between thrombus and lumen, and also between thrombus and brain tissue. The feasibility of using MRBTI for thrombus volume measurement was also explored.

## Results

With effectively suppressed blood signal**,** MRBTI correctly identified 113 out of 116 segments with proven CVT with a sensitivity of 97.4%. In 527 out 531 segments, CVT was ruled out correctly with a specificity of 99.3%. Quantification of thrombus volume was successfully conducted in all patients with CVT, and mean volume of thrombus was 10.5 ± 6.9 cc.

## Conclusions

The current findings support that MRBTI allows direct selective visualization of thrombus as opposed to indirect detection of venous flow perturbation and can be used as a promising first line diagnostic imaging tool.

**Figure 1:**
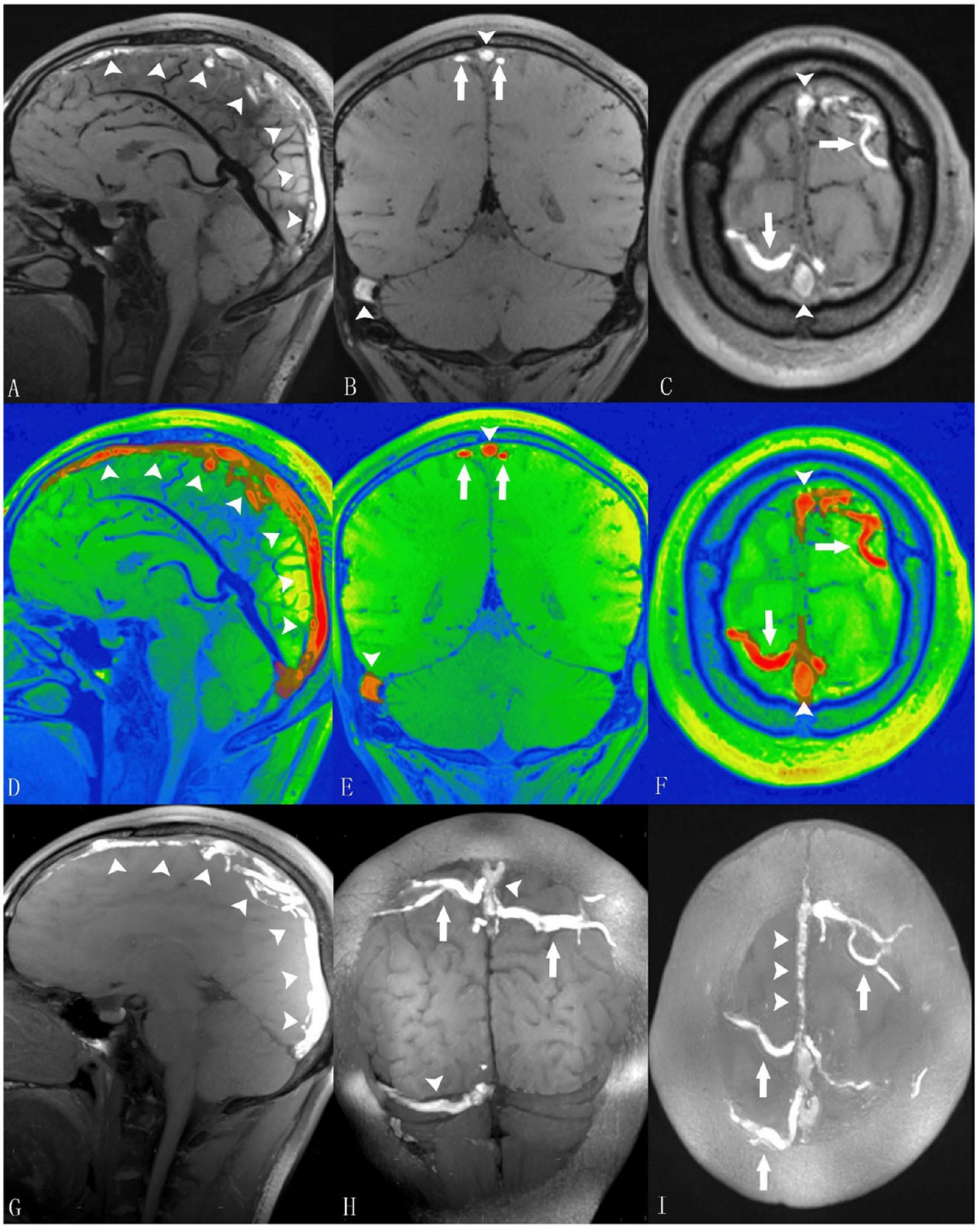
**MRBTI of a 27-year-old male patient with sub-acute CVT**. A-C: MRBTI demonstrated hyper-intense signal intensity in the superior sagittal sinus (arrowheads), the right transverse and sigmoid sinuses (arrowheads), and the cortical veins (arrows) suggesting intraluminal thrombus formation. D-F: All thrombi semi-automatically outlined by software based on their high signal contrast were rendered with red color and volume was 21.5 cc. G-I: sagittal, coronal and axial sections of maximum intensity projection (MIP) reformations of MRDTI better depicted the thrombosed segments with hyper-intense signals.

